# A Novel, “Double-Clamp” Binding Mode for Human Heme Oxygenase-1 Inhibition

**DOI:** 10.1371/journal.pone.0029514

**Published:** 2012-01-19

**Authors:** Mona N. Rahman, Jason Z. Vlahakis, Dragic Vukomanovic, Wallace Lee, Walter A. Szarek, Kanji Nakatsu, Zongchao Jia

**Affiliations:** 1 Department of Biomedical and Molecular Sciences, Queen's University, Kingston, Canada; 2 Department of Chemistry, Queen's University, Kingston, Canada; University of South Florida College of Medicine, United States of America

## Abstract

The development of heme oxygenase (HO) inhibitors is critical in dissecting and understanding the HO system and for potential therapeutic applications. We have established a program to design and optimize HO inhibitors using structure-activity relationships in conjunction with X-ray crystallographic analyses. One of our previous complex crystal structures revealed a putative secondary hydrophobic binding pocket which could be exploited for a new design strategy by introducing a functional group that would fit into this potential site. To test this hypothesis and gain further insights into the structural basis of inhibitor binding, we have synthesized and characterized 1-(1*H*-imidazol-1-yl)-4,4-diphenyl-2-butanone (**QC-308**). Using a carbon monoxide (CO) formation assay on rat spleen microsomes, the compound was found to be ∼15 times more potent (IC_50_ = 0.27±0.07 µM) than its monophenyl analogue, which is already a potent compound in its own right (**QC-65**; IC_50_ = 4.0±1.8 µM). The crystal structure of hHO-1 with **QC-308** revealed that the second phenyl group in the western region of the compound is indeed accommodated by a definitive secondary proximal hydrophobic pocket. Thus, the two phenyl moieties are each stabilized by distinct hydrophobic pockets. This “double-clamp” binding offers additional inhibitor stabilization and provides a new route for improvement of human heme oxygenase inhibitors.

## Introduction

**Figure 1:**
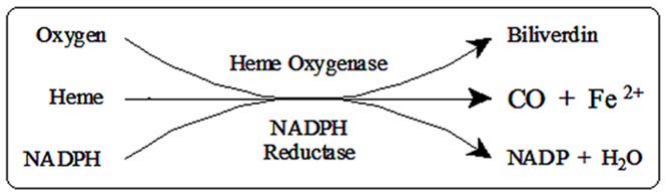
The oxidative degradation of heme in the carbon monoxide/heme oxygenase (CO/HO) pathway.

The heme oxygenase (HO) system comprises two active isozymes (HO-1 and HO-2) which are involved in the regioselective, oxidative cleavage of heme at the α-meso carbon. Cleavage results in the release of equimolar amounts of the gasotransmitter carbon monoxide (CO), ferrous iron (Fe^2+^), and biliverdin, the latter of which is reduced further by biliverdin reductase to form bilirubin [1]–[3] (Figure 1). A third isozyme, HO-3, has been deemed inactive despite exhibiting ∼90% sequence identity with HO-2 [4]. Of the two active isozymes, HO-1 is a ∼32 kDa stress protein found to be predominantly expressed in the spleen and inducible by a variety of stimuli including heat shock, heavy metals, heme and reactive oxygen species (ROS). The constitutive HO-2, a ∼36.5-kDa protein, is widely distributed with its highest concentration in the brain and testes.

The HO reaction comprises ∼85% of the CO produced in humans under normal physiological conditions. CO has been found to be one of the most important gasotransmitters in the body, with evidence demonstrating regulatory involvement in anti-inflammatory, antiapoptotic, antiproliferative and vasodilatory effects [5]–[8]. Both biliverdin and bilirubin act to scavenge free radicals such as superoxide, peroxyl radicals and peroxynitrite, making them both powerful antioxidants [9]–[11]; indeed, bilirubin has been shown to provide protection against oxidative stress-induced pathologies [5]. In general, the HO system has demonstrated a protective role against many diseases including ischaemia-reperfusion injury and endotoxic shock, as well as against graft rejection [12]. HO-1 has been found to do be upregulated in several cancers, including pancreatic and prostate, and in response to several anticancer treatments [13]–[15].

The development of isozyme-selective HO inhibitors should provide powerful pharmacological tools in understanding the HO/CO system, elucidating the mechanisms underlying its physiological effects, as well as opening the door to potential therapeutic applications. Historically, the major inhibitors used to date have been the metalloporphyrins (e.g. tin/zinc/chromium protoporphyrin) which act as competitive inhibitors due to their structural similarity to the heme substrate. However, since heme is essential for a number of biologically relevant enzymes, including cytochrome P450, nitric oxide synthase (NOS) and soluble guanylyl cyclase (sGC), the use of structural analogues to heme is limited due to their lack of selectivity. Indeed, this has led to some concerns about the interpretation of results and resultant conclusions from previous studies of the HO/CO system [16]–[18]. It should be noted, however, that studies have also demonstrated that some of these metalloporphyrins do maintain selectivity against HO *in vitro* if used within a defined concentration range; however, this is restricted to chromium mesoporphyrin IX at concentrations up to 5 µM [19]. Thus, there is an obvious and pressing need to develop isoselective HO inhibitors.

**Figure 2:**
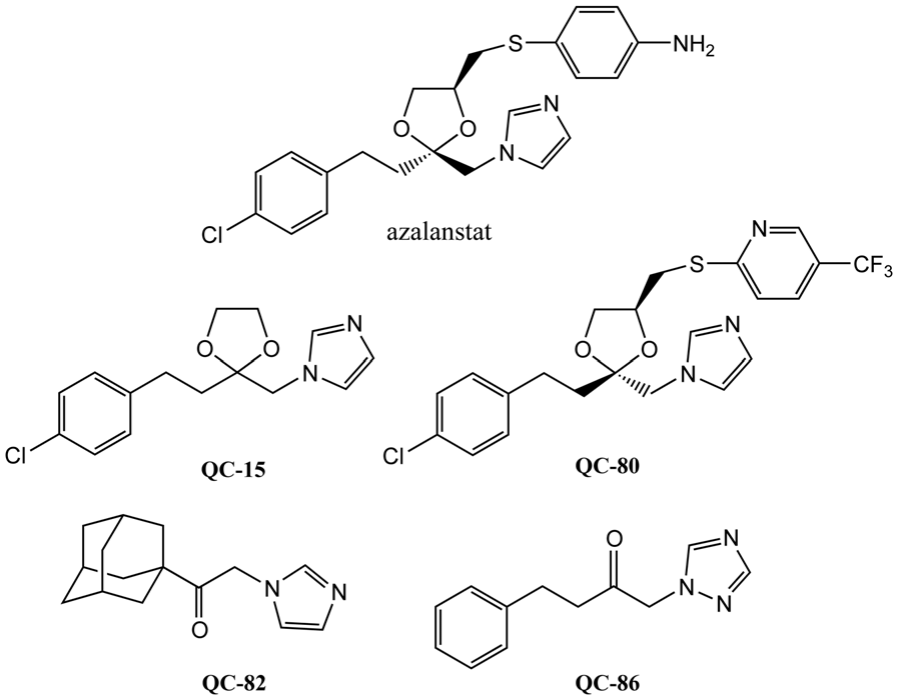
Structures of azalanstat and QC-xx compounds used for crystallization studies.

Our laboratory has been focused on a program aimed at the design of novel, non-porphyrin based, isozyme-selective HO inhibitors based on the structure of (2*S*, 4*S*)-2-[2-(4-chlorophenyl)ethyl]-2-[(1*H*-imidazol-1-yl)methyl]-4-[((4-aminophenyl)thio)methyl]-1,3-dioxolane (azalanstat, Figure 2), which was discovered as a potent and selective inhibitor of HO [20]. This program has resulted in the discovery of several novel, azole-based compounds, deemed **QC-xx**, which selectively and non-competitively inhibit HO without affecting NOS or sGC, except at high millimolar concentrations, and do not induce expression of NOS, sGC, or HO-1 [21]. Recently, we have also discovered that replacing the imidazole by triazole or tetrazole greatly decreases the ability of this series of compounds to inhibit the cytochrome P450 family of drug metabolizing enzymes [22]. We have been successful in identifying several compounds as potent and selective inhibitors of HO-1 [21], [23]–[27]. Preliminary data show some of these compounds to be able to attenuate the tumour size and metastases of breast cancer cells (Dercho *et al.*, unpublished results). Moreover, several of our compounds are already being used by other research groups as tools to dissect HO function [28].

As part of our program to “build a better inhibitor” we have complemented functional analyses with structural studies of complexes formed between human HO-1 and various **QC-xx** complexes (Figure 2) using X-ray crystallography (reviewed in [29]). We have successfully crystallized complexes with 1-(adamantan-1-yl)-2-(1*H*-imidazol-1-yl)ethanone (**QC-82**) [30], 4-phenyl-1-(1,2,4-1*H*-triazol-1-yl)-2-butanone (**QC-86**) [27], and (2*R*, 4*S*)-2-[2-(4-chlorophenyl)ethyl]-2-[(1*H*-imidazol-1-yl)methyl]-4-[((5-trifluoromethylpyridin-2-yl)thio)methyl]-1,3-dioxolane (**QC-80**) [31]. These structures, in addition to that of 2-[2-(4-chlorophenyl)ethyl]-2-[1*H*-imidazol-1-yl)methyl]-1,3-dioxolane (**QC-15**) with rat HO-1 [32], have enabled the identification of key features required for binding to hHO-1 as well as the mechanism underlying its inhibition, and insights toward the design of more effective inhibitors. In general, these azole-based inhibitors exhibit a common binding and inhibition mode. The azole moiety in the eastern region serves as an anchor which coordinates with the iron atom through nitrogen 3 to form a hexacoordinate heme. Replacement of imidazole with a triazole anchor may provide additional stability by the formation of a hydrogen bond between the additional nitrogen atom and the protein's distal helix. The linker in the central region attaches this anchor to a hydrophobic group in the western region which fits into a hydrophobic pocket in the distal region of the heme-binding pocket. While hydrophobic and stacking interactions contribute to the stability of this region, halogenation is also accommodated in this western region putatively due to the presence of Met34 in the pocket or, alternatively, greater and more points of contact within the pocket. The distal helix of the enzyme has an inherent flexibility which allows for a range of bulky functional groups without displacing the heme moiety or the catalytically critical Asp140. The proximal helix was also found to have some degree of flexibility; interaction of **QC-80**, containing a large northeastern substituent, induces a conformational change to “unkink and extend” the helix and induce the formation of a peripheral hydrophobic pocket to accommodate this group. As a result, binding of the **QC-xx** inhibitors to the distal side of the heme binding pocket results in the disruption of an ordered hydrogen bond network involving Asp140 and displacement of a catalytically critical water molecule, to inhibit heme oxidation.

**Figure 3:**
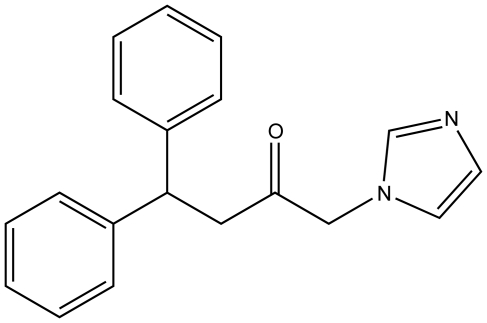
Structure of 1-(1*H*-imidazol-1-yl)-4,4-diphenyl-2-butanone (QC-308). **QC-65** lacks the second phenyl moiety.

Previously, one of our crystal structures of inhibitor-complexed hHO-1 revealed a putative, proximal secondary hydrophobic pocket not apparent in other complex or native structures [30]. To explore the potential of this site, we developed a new design strategy to create compounds which would occupy both hydrophobic pockets simultaneously in order to increase potency. Thus, we have synthesized and characterized 1-(1*H*-imidazol-1-yl)-4,4-diphenyl-2-butanone (**QC-308**), the diphenyl analogue of one of our most potent inhibitors, (4-phenyl-1-(1*H*-imidazol-1-yl)-2-butanone (**QC-65**; IC_50_ = 4.0±1.8 µM) [23], which contains an additional phenyl group in the western region of the compound, in contrast to the usual single hydrophobic moiety found in most of this series (Figure 3). Notably, since **QC-65** is already one of the most potent inhibitors, further improvement in potency was very challenging and required a new strategy such as the one exploited to create **QC-308**. The X-ray crystal structure of the inhibitor-hHO-1 complex definitively demonstrates the presence of a secondary hydrophobic pocket which acts to stabilize the second phenyl moiety of this structure in addition to the distal hydrophobic pocket common to previous structures. As a result, the two phenyl moieties each fit into separate hydrophobic pockets. This “double-clamp” binding mode, thus, most likely accounts for the increased potency of this inhibitor by providing additional stabilization, thereby providing for improvement of human heme oxygenase inhibitors.

## Results and Discussion

### Synthesis and Functional Characterization of QC-308

**Figure 4:**
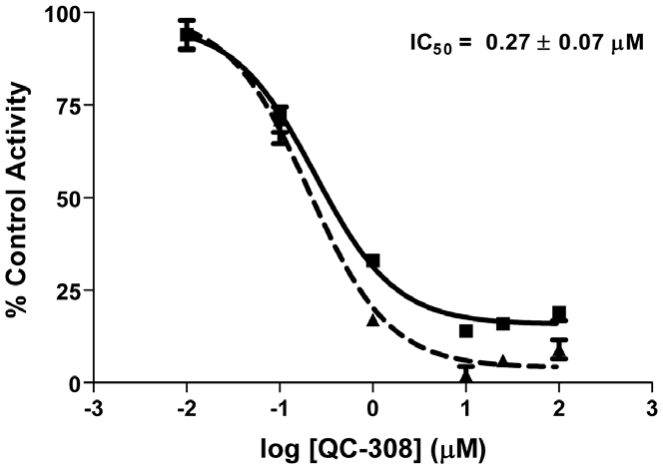
Inhibition of HO-1 activity by QC-308. Enzyme activity was determined by measuring the CO produced in 15 min from 50 µM methemalbumin using 0.5 mg/mL rat spleen microsomes. The IC_50_ was determined by nonlinear regression using GraphPad Prism version 4. Curves represent two independent trials, with each performed in duplicate.

In this study, we report the synthesis and characterization of 1-(1*H*-imidazol-1-yl)-4,4-diphenyl-2-butanone (**QC-308**), the diphenyl analogue of one of our most potent HO inhibitors, **QC-65** [23]. The synthesis involved bromination of commercially available 4,4-diphenyl-2-butanone to afford 1-bromo-4,4-diphenyl-2-butanone, followed by nucleophilic displacement of the bromo group by imidazole to give 1-(1*H*-imidazol-1-yl)-4,4-diphenyl-2-butanone and conversion into its hydrochloride **QC-308**. One of our initial crystal structures of inhibitor-complexed hHO-1 revealed a putative, secondary hydrophobic pocket proximal to the distal pocket, not observed in other native or complexed structures [30]. In that previous analysis, it was suggested that this secondary pocket was induced upon binding of inhibitor as it was not apparent in the high resolution native structure. At the time, it was postulated that it may accommodate the bulky group in the thio-(4-aminophenyl) region of azalanstat which we later refuted by the observation of the inducible binding mode of hHO-1 with **QC-80** [31]. Thus, to exploit this putative interaction, we modified our strategy and **QC-308** was designed to contain two phenyl groups which would occupy both hydrophobic pockets simultaneously (Figure 3). Initial screening of **QC-308** using a CO formation assay with rat spleen microsomal fractions gave an IC_50_ of 0.27±0.07 µM (Figure 4), ∼15-fold more potent than its monophenyl analogue (IC_50_ = 4.0±1.8 µM, [23]).

**Figure 5:**
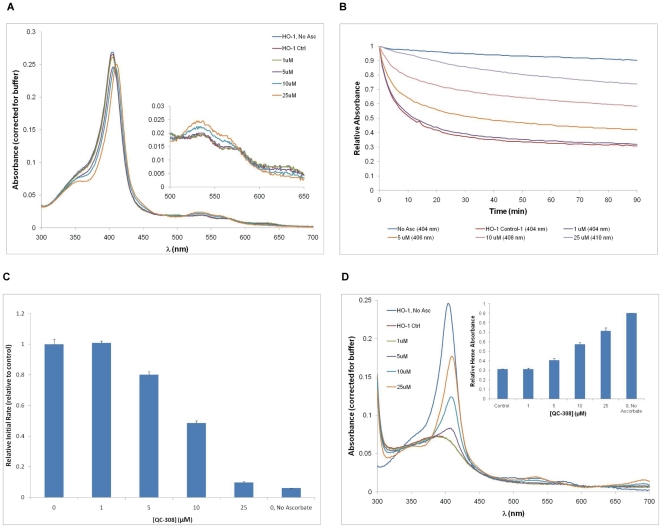
Spectral analysis of QC-308 binding to hHO-1. (A) Heme-conjugated hHO-1 (10 µM) in 20 mM potassium phosphate (pH 7.4) was incubated with increasing concentrations of **QC-308** at room temperature. Absorbances were measured over a range of 300-700 nm at intervals of 1 nm, and values were corrected for buffer (20 mM potassium phosphate, pH 7.4). The assays were performed in duplicate and the values averaged. The Soret peak gradually shifted from 404 to 410 nm with increasing concentrations of **QC-308**. Secondary peaks centered at 535 and 560 nm were amplified with increasing concentrations of inhibitor, while a third minor peak at 630 nm decreased until no longer detectable at high inhibitor concentrations (B) Heme degradation rates in the presence of **QC-308**. Heme degradation was subsequently initiated by the addition of 1 mM l-ascorbic acid and allowed to proceed for 90 min at room temperature. Absorbances were measured at 404, 406, 408 and 410 nm at 1 min intervals and normalized to the initial absorbance (t = 0) for the respective condition. Graph is representative of one replicate for each condition at the wavelength of its Soret peak (indicated in legend). (C) Initial rates were determined for each condition over a period of 1 min (from t = 2–3 min) at the wavelength corresponding to its respective Soret peak as indicated in the legend in (B). For each replicate, values were normalized to the respective control condition, and subsequently averaged. Parallel reactions were also performed for heme-conjugated hHO-1 in the absence of both inhibitor and electron donor (l-ascorbic acid) as a negative control (i.e., no oxidative degradation). (D) Spectral analysis following heme degradation. Absorbances were measured and analyzed as described in (A). Heme degradation corresponded to the disappearance of the Soret peak. Increasing concentrations of inhibitor resulted in increased attenuation of the loss of the Soret peak as well as of the secondary peaks at 535 and 560 nm but the appearance of a peak at 699 nm. Inset depicts the fraction of heme still undegraded after the 90 min reaction, relative to that present at t = 0 for each condition.

Binding of **QC-308** to the truncated, recombinant hHO-1 protein used for crystallization was also assessed spectrally. As seen with our previous azole-based inhibitors [30], [33], in the absence of inhibitor, the heme-bound hHO-1 showed a characteristic spectrum with a Soret peak of 404 nm. In the presence of increasing amounts of inhibitor (1–25 µM), the peak was red-shifted up to 410 nm with a more prominent shoulder centred at 355 nm (Figure 5A). The increases in secondary peaks at 535 and 560 nm with a decrease at 630 nm were also similar to what was previously observed with this type of inhibitor [30], thus implying a change in the heme environment and putative binding of the inhibitor. Heme degradation in the presence of increasing amounts of inhibitor was initiated by the addition of l-ascorbic acid, and followed for 90 minutes. Heme absorbance was half-maximal by 11–12 minutes in the absence of inhibitor, which was retarded with increasing amounts of **QC-308** (Figure 5B). Initial rates were determined for each condition by calculating the rate of change of absorbance over the initial linear portion of the graph (between 2–3 min), according to its respective Soret peak. At a concentration of 25 µM the Soret peak had shifted to 410 nm, and the initial rate had decreased to 9.8±0.5% of the control (Figure 5C). A full spectral analysis was also performed after the 90 minutes to measure the attenuation of heme degradation by **QC-308**. With increasing amounts of inhibitor, there was a concomitant increase in the height of the Soret peak indicating the amount of intact heme remaining; at 25 µM, 71.6±3.0% of the original heme was undegraded *cf*. 31.1±0.3% for the control (Figure 5D).

There appears to be a discrepancy in the potency of this compound between the CO formation assay on spleen microsomes and the optical assay with recombinant protein. Notably, the initial rate was decreased to ∼50% by 10 µM i.e. a 1∶1 enzyme∶inhibitor molar ratio which is much higher than the IC_50_ (0.27 µM) determined with the microsomal preparation. Indeed, this discrepancy was observed in previous studies comparing results from spleen microsomes and this recombinant truncated hHO-1 which revealed that the longer, native form of HO-1 was more susceptible to inhibition than the truncated form [34].

### Crystallization and Structure Determination of hHO-1 in Complex with QC-308

Similar to previous co-crystallization studies with the azole-based HO-inhibitors, binding of **QC-308** to hHO-1 was confirmed prior to setting up co-crystallization trials by spectral analyses. Protein was incubated with **QC-308** at a 1∶3 molar ratio, respectively, resulting in a shift in the Soret peak from 404 nm to 410 nm, similar to what had been observed previously [30], [31], thus verifying binding. Co-crystallization trials were based on previous conditions and resulted in crystals very similar to those obtained previously from both native and hHO-1 in complex with the previous azole-based inhibitors. Diffraction data were obtained to a resolution of 2.85 Å. In contrast to previous native [35]–[37] and complex structures of hHO-1 [26], [27], [29]–[31], the data were assigned to the *P*2_1_2_1_2_1_ space group with larger unit cell dimensions. Interestingly, this is similar to that found for the hHO-2 crystal structure [38]. Molecular replacement was carried out as previously described using the native heme-conjugated hHO-1 complex (PDB code 1N3U, A chain) as the initial probe. Two unambiguous solutions, corresponding to the two molecules in the asymmetric unit, were obtained (LLG = 2315) and the position of the inhibitor was clear from the resultant *F_o_-F_c_* map following initial refinement. The structure was refined to an *R* of 0.223 and *R_free_* of 0.264. A total of 26 water molecules were added to the structure as well as one molecule of 1,6-hexanediol (Figure S1). The Ramachandran plot demonstrated no residues in the disallowed region. Diffraction and final refinement statistics are given in Table 1.

**Table 1:** Diffraction and refinement statistics^a^.

Space group	*P*2_1_2_1_2_1_
*a* (Å)	54.61
*b* (Å)	74.98
*c* (Å)	115.28
β (°)	90.000
Molecules in the asymmetric unit	2
Solvent content (%)	41.65
Mosaicity (°)	0.51
Resolution range (Å)	20–2.85
Total reflections	48116 (2557)
Unique reflections	11070 (570)
Completeness (%)	95.6% (96.9%)
I/σ	20.81 (3.82)
bR*_merge_*	7.2% (56.1%)
Average redundancy	4.35 (4.46)
Refinement statistics	
Sigma cutoff for refinement	None
cR*_crys_*	0.22312
cR*_free_*	0.26377
Number of reflections used	10516
Number of reflections in test set	554
Number of non-hydrogen atoms used in refinement	3662
RMSD bond lengths (Å)	0.006
RMSD bond angles (°)	0.910
Number of water molecules	26
Ramachandran Plot	
Most favoured (%)	93.3
Additional allowed (%)	6.7
Generously allowed (%)	0.0
Disallowed regions (%)	0.0

a Values in parentheses are for the outermost shell (2.91-2.85 Å).

b R_merge_ = Σ|I_obs_−<I>|/ΣI_obs_, where I_obs_ is the intensity measurement and <I> is the mean intensity for multiply recorded reflections.

c R_cryst_ and R_free_ = Σ|F_obs_−F_calc_|/Σ|F_obs_| for reflections in the working and test sets, respectively.

The two molecules in the asymmetric unit (A and B) were virtually superimposable with a Cα RMSD of 0.391 Å (upon alignment of all atoms). The greatest discrepancy was observed in the distal helix (Gly144: 1.286 Å) (Figure S2), which is not surprising given its inherent flexibility [35]. Density corresponding to a molecule of 1,6-hexanediol was also only observed in molecule A. The position of this molecule was remote from the inhibitor binding site and was, thus, deemed irrelevant to the protein's interaction with **QC-308**. In general, the density associated with molecule A was more disordered and less complete than that associated with molecule B. As such, all further analyses will be focused on molecule B.

**Figure 6:**
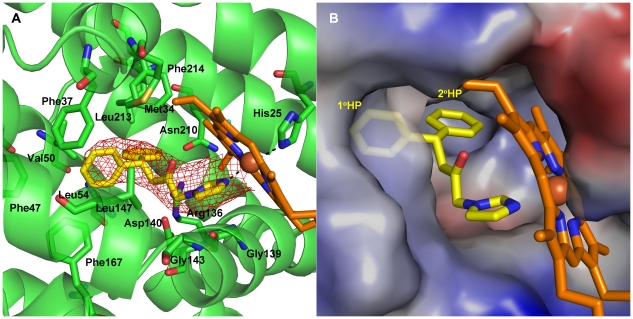
Crystal structure of heme–conjugated hHO-1 in complex with QC-308 at 2.85 Å resolution. (A) Ribbon diagram of the inhibitor binding site. Heme (orange) and **QC-308** (yellow) are depicted as stick models. An omit map (*F_o_-F_c_*) contoured at 2σ is superimposed. Dashed lines indicate coordination of imidazole nitrogens of **QC-308** and His25 with the heme Fe. Residues involved in inhibitor binding are indicated. (B) Electrostatic surface potentials revealing the presence of two distal hydrophobic pockets (1° HP and 2° HP) which accommodate the two phenyl groups of **QC-308**: a “double-clamp”. Dashes indicate coordination of the imidazole group with the heme Fe. Blue and red colours indicate positive and negative electrostatic potentials, respectively, as calculated using PyMOL [60].

The overall structure of hHO-1 in complex with **QC-308** was similar to previous structures determined for the protein with the azole-based inhibitors, generally following the common binding mode already established for most of this type of inhibitor (reviewed in [29]). In brief, the inhibitor binds to the distal side of heme in the heme-binding pocket with the imidazolyl group serving as the sixth ligand of coordination with the heme iron i.e. the anchor for the binding (Fe–imidazolyl distance = 2.1 Å) (Figure 6A). The western region of the inhibitor fits into the hydrophobic pocket which extends back towards the distal side of the heme binding pocket with the distal helix shifting outward to accommodate this region. Alignment with the “closed”, more active, form of the native holoenzyme (PDB Code #1N45) shows a maximal shift outward of 2.349 Å (Gln145) in this distal helix. Interestingly, the distal helix does not shift as much as was observed in binding the **QC-82** compound (PDB Code # 3CZY) which contains the bulky adamantanyl substitutent in the western region, despite the larger area taken up by the diphenyl moiety; the Gly144 residue is shifted 2.972 Å further with **QC-82** than the current complex structure. Moreover, comparison with the complex of hHO-1 with **QC-80**, which contains a bulky substituent in the northeastern region, indicated that the induced shift of the proximal helix observed in that complex resulting in a novel, proximal hydrophobic pocket to accommodate the bulky 5-trifluoromethylpyridin-2-yl group [31] is also not necessary (Figure S2). In fact, the **QC-308** compound seems to “slide” easily into its binding site with relatively little conformational change to the native structure.

Examination of the electrostatic surface representation of the hHO-1–**QC-308** structure shows the presence of the distal hydrophobic pocket which accommodates one of the two phenyl groups (Figure 6B). Moreover, the second phenyl moiety fits comfortably into a secondary proximal hydrophobic pocket, very similar to but larger than that observed previously. Analysis of the protein residues within van der Waals distance of the inhibitor (Table S1) indicates that metal coordination and hydrophobic interactions stabilize its binding. No hydrogen bond networks, seen to be involved in the stabilization of some previous compounds, were apparent in this structure. Thus, it appears that the two hydrophobic pockets serve as a “double-clamp” to stabilize the two phenyl groups in the western region of this compound and increase the stability of the complex, making for a more potent inhibitor than those previously designed.

### Isozyme-selectivity of QC-308

**Figure 7:**
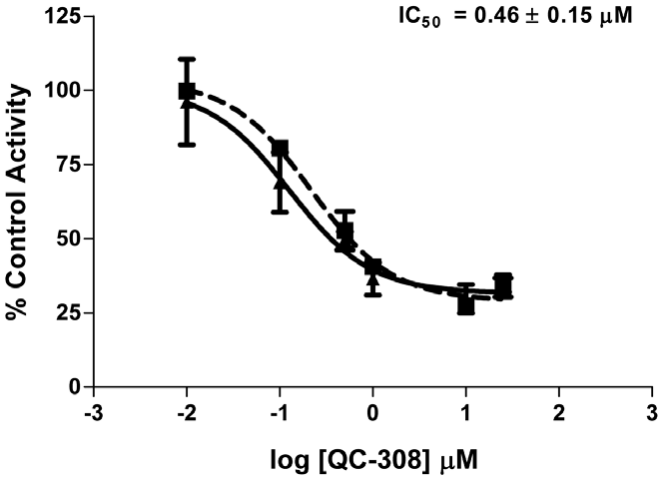
Inhibition of HO-2 activity by QC-308. Enzyme activity was determined by measuring the CO produced in 15 min from 50 µM methemalbumin using 0.5 mg/mL rat brain microsomes. Calculations were performed as described for Figure 4.

**Figure 8:**
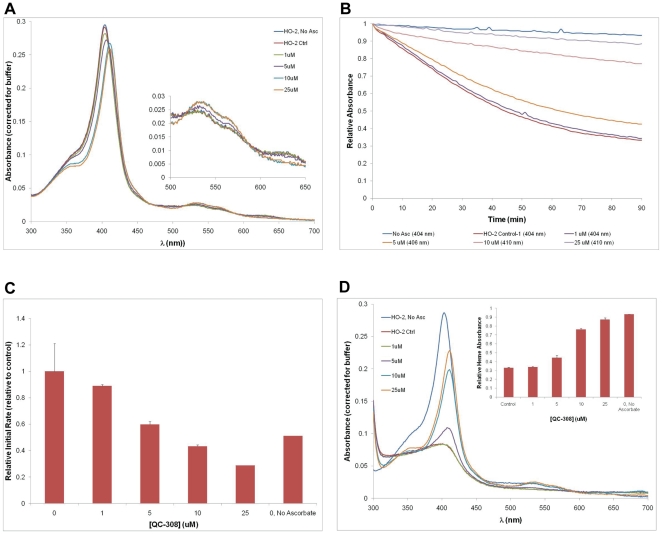
Spectral analysis of QC-308 binding to hHO-2. Analyses were done in parallel to those in Figure 5.

The **QC-308** compound was also screened for inhibitory activity against HO-2 using rat brain microsomal fractions. Using the CO formation assay, an IC_50_ of 0.46±0.15 µM was obtained (Figure 7), comparable to that of HO-1. Parallel experiments were also performed using a recombinant hHO-2 truncation derivative (1–264 amino acids with an N-terminal His–tag) to allow comparison with the hHO-1 truncated protein (Figure 8). Interestingly, the spectral changes upon addition of **QC-308** were similar to those seen for hHO-1, implying a similar change in the heme environment (Figure 8A). It should be noted that, in this system, the rate of heme degradation for the truncated hHO-2 (0.013±0.012 pmol CO/min) was found to be less than that of hHO-1 (0.060±0.002 pmol CO/min), as observed by the shallower curves; heme absorbance was half-maximal by 42–44 minutes (*cf.* 11–12 minutes for hHO-1) (Figure 8B). Increasing the inhibitor concentration also attenuated the degradation of heme as observed by the heights of the Soret peaks measured after the reaction; 25 µM of **QC-308** resulted in 87.2±1.9% of the original heme still undegraded *cf.* 32.8±0.7% for the control. Interestingly, the potency of inhibition with this truncation derivative is also discrepant relative to the CO formation assay. It should be noted that in our previous comparisons of native versus recombinant HO-2, the results were more comparable. However, the recombinant protein used in that study was a GST-fusion derivative which also contained a Cys127Ala mutation which disrupts HRM3, one of HO-2's heme regulatory motifs (HRMs).

Previous analyses of **QC-65** had revealed IC_50_ values of 4.0±1.8 µM and 11.3±4.7 µM against HO-1 and HO-2, respectively, using the CO formation assay with rat spleen and brain microsomes [23]. Isoselectivity was seen when the central ketone group of **QC-65** was changed to a dioxolane (**QC-57**; 2-[2-phenylethyl]-2-[(1*H*-imidazol-1-yl)methyl]1,3-dioxolane) with IC_50_'s of 0.7±0.4 µM and >100 µM for HO-1 and HO-2, respectively [23]. It would be interesting to see if a diphenyl analogue of this compound could further improve potency while maintaining isoselectivity.

**Figure 9:**
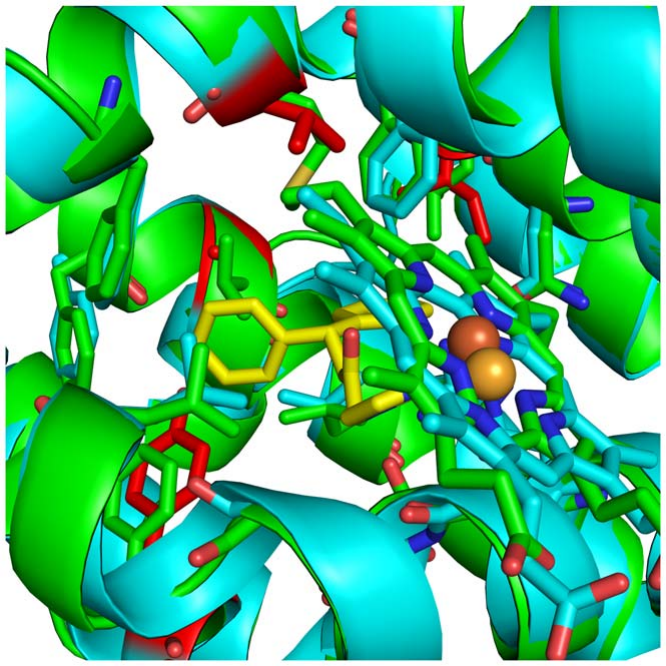
Ribbon diagram showing the structural alignment of hHO-2 (cyan) with the hHO-1–QC-308 complex (green). Residues involved in **QC-308** binding are depicted as stick models, as are heme and **QC-308** (yellow). The two structures are virtually identical in this catalytic core. Residues of hHO-2 which differ amongst the contact residues of the hHO-1 inhibitor binding site are depicted in red. Structural alignments were performed using “Superpose” in CCP4 [54], [58].

In order to try and determine the basis of isoselectivity, we turned again to structural analyses. The crystal structure of a truncated derivative of hHO-2 (1–264; Cys127Ala) has been determined (PDB Code #2QPP) [38]. Alignment of this structure of the native hHO-2 holoenzyme with that of the hHO-1–**QC-308** complex confirmed that the catalytic cores of these two enzymes are structurally conserved [38] with an RMSD of 0.874 Å over the 202 aa alignment length. Closer inspection of the residues involved in **QC-308** interaction in hHO-1 showed no significant changes in hHO-2 which may account for the isozyme-selective behaviour (Figure 9) seen when the ketone is changed to a dixolane group. Indeed, of the contact residues, only four differences were seen between the two isozymes: Phe167Tyr, Val50Ala, Met34Val, and Leu213Ile. The Cys127 residue comprising HRM3 (mutated to Ala in the HO-2 structure) is remote from the heme/inhibitor binding site implying that it also may not be involved in attributing selectivity on its own. Moreover, it is not immediately apparent as to why the two recombinant proteins would have such different catalytic rates in the optical assay.

The greatest sequence divergence between HO-1 and HO-2 lies within the C-terminal domain which contains a membrane-binding domain, with a second region around Cys127 in HO-2 (i.e. HRM3) [38]. Studies comparing recombinant full-length HO-1 protein and a soluble 30 aa truncated protein indicated that the C-terminal 23 amino acids are essential for maximal activity (2–5× when measuring bilirubin formation) [39], [40]. The C-terminal domain is thought to mediate the formation of a high-affinity complex with cytochrome P-450 reductase, as well as, membrane incorporation, thus resulting in increased activity [41]. The C-terminus seems to have a regulatory role in both of these isozymes. In HO-1, cleavage of 52 aa at the C-terminus affects nuclear import, leading to activation of genes associated with protection against oxidative stress [42]. In HO-2, heme regulatory motifs (HRM1 and HRM2) are located in this region and act as a thiol/disulfide redox switch to modulate heme affinity depending on the oxidation state of the environment. Early studies suggested that these HRMs are additional heme binding motifs [43]–[45]. However, more recent studies have shown that the HRMs affect the heme K*_d_*, but not the k_cat_ for heme degradation, without binding additional heme moieties [46], [47]. Moreover, spectral studies suggest Cys265 of HRM1 competes with His45 as an axial ligand to ferric heme bound to HO-2 under reducing conditions [48]. Unfortunately, the crystal structures of both human HO-1 and HO-2 are of truncated proteins which lack this region. Furthermore, in the HO-2 structure, both the membrane binding domain and two heme regulatory motifs (HRM1 and HRM2) are missing while HRM3 has been removed by a Cys127Ala mutation [38]. Thus, structural information regarding the full-length isozymes will be crucial in elucidating isoselectivity of the **QC-xx** compounds as well the basis behind the lack of selectivity for **QC-308** and **QC-65**, as opposed to **QC-57**.

### Conclusions

The binding of **QC-308** to hHO-1 reveals a novel “double-clamp” binding mode. The presence of a secondary hydrophobic pocket provides an additional site of stabilization for the second phenyl group in this **QC**-analogue which may account for the ∼15-fold increase in potency for this inhibitor. This information may be utilized as a basis to modify isozyme-selective inhibitors to increase potency while maintaining isoselectivity. It is clear, however, that structural information regarding the full-length isozymes will be crucial in elucidating isoselectivity of the **QC-xx** compounds.

## Materials and Methods

### Ethics Statement

All animals were cared for in accordance with the principles and guidelines of the Canadian Council on Animal Care, and experimental protocols were approved by the Queen's University Animal Care Committee.

### Synthesis of QC-308

#### General

Flashcolumn chromatography was performed on Silicycle silica gel (230–400 mesh, 60 Å). Analytical thin-layer chromatography was performed on glass-backed pre-coated Silica Gel 60 F_254_ plates (Silicycle), and the compounds were visualized by UV illumination (254 nm). Melting points were measured on a Mel-Temp II apparatus and are uncorrected. ^1^H and ^13^C NMR spectra were recorded on a Bruker Avance 400 spectrometer in CDCl_3_ or CD_3_OD. The chemical shifts are reported in δ (ppm) relative to tetramethylsilane [49]. The compounds synthesized were deemed >95% pure by ^1^H NMR analysis. High-resolution ESI mass spectra were recorded on an Applied Biosystems/MDS Sciex QSTAR XL mass spectrometer with an Agilent HP1100 Cap-LC system. Samples were run in 50% aqueous MeOH at a flow rate of 6 µL/min. High-resolution EI mass spectra were recorded on a Waters/Micromass GC-TOF instrument.

#### Preparation of 1-bromo-4,4-diphenyl-2-butanone

(4,4-Diphenyl-2-butanone was obtained from Alfa Aesar, Ward Hill, MA, USA.) Under an atmosphere of nitrogen, 4,4-diphenyl-2-butanone (1248 mg, 5.56 mmol, 1 equiv) was dissolved in methanol (10 mL) and a solution of bromine (0.29 mL, 889 mg, 5.56 mmol, 1 equiv) in methanol (7 mL) was added dropwise at RT. The mixture was stirred at RT for 6 h, an aqueous solution of sodium thiosulfate (0.3 M, 10 mL) was added, and the mixture concentrated. To the residue was added brine, and the mixture extracted with ethyl acetate (3×). The combined organic extracts were washed with brine (2×), dried over sodium sulfate, then concentrated. High-vacuum drying left the crude product (1745 mg) as a brown oil; ^1^H NMR (400 MHz, CDCl_3_): *δ* 3.43 (d, *J* = 7.6 Hz, 2H), 3.75 (s, 2H), 4.63 (s, 1H), in addition to signals attributed to starting material and the 3-bromo isomer; ^13^C NMR (100 MHz, CDCl_3_): *δ* 34.8, 46.0, 46.3, 126.8, 127.8, 128.8, 143.4, 200.3; HRMS (EI) [M]^+^ Calculated for C_16_H_15_BrO: 302.0306. Found: 302.0286.

#### 1-(1H-Imidazol-1-yl)-4,4-diphenyl-2-butanone hydrochloride (QC-308)

Under an atmosphere of nitrogen, a sample of the crude 1-bromo-4,4-diphenyl-2-butanone (836 mg, 2.76 mmol, 1 equiv) was dissolved in *N*,*N*-dimethylformamide (11 mL) and to this solution was added imidazole (752 mg, 11.05 mmol, 4 equiv) at RT. The mixture was stirred at 80°C for 24 h, then cooled to RT. Water was added and the mixture extracted with ethyl acetate. The organic extract was washed sequentially with a solution of sodium bicarbonate in brine (3×), and brine, dried over sodium sulfate, then concentrated. The residue was purified by flash column chromatography on silica gel (ethyl acetate as eluent, product *R_f_* ∼0.2) to give the free base (144 mg, 0.50 mmol, 18%). To a solution of the free base in warm 2-propanol (2 mL) was added a solution of 37% aqueous HCl (60 mg, 0.61 mmol, 1.2 equiv) in 2-propanol (2 mL). The mixture was concentrated and dried under high vacuum, leaving the product (151 mg, 0.46 mmol, 17%) as a beige solid; mp 218–220°C; ^1^H NMR (400 MHz, CD_3_OD): *δ* 3.48 (d, *J* = 7.6 Hz, 2H), 4.61 (t, *J* = 7.4 Hz, 1H), 5.27 (s, 2H), 7.16–7.20 (m, 2H), 7.24–7.34 (m, 9H), 7.52 (s, 1H), 8.75 (s, 1H); ^13^C NMR (100 MHz, CD_3_OD): *δ* 46.5, 46.9, 58.3, 120.5, 124.5, 127.6, 128.8, 129.7, 137.6, 145.1, 201.1; HRMS (ESI) [M-Cl]^+^ Calculated for C_19_H_19_N_2_O: 291.1497. Found: 291.1490.

#### HO Activity Assay

HO activity in rat spleen and microsomal fractions was determined by quantifying the CO formed from the degradation of methemalbumin (heme complexed with albumin) [50], [51] as described previously [30].

#### Expression and Purification of hHO-1 and hHO-2

A truncated, soluble version of hHO-1 containing 233 amino acids (hHO1-t233) was previously utilized successfully to solve the high-resolution crystal structure of native hHO-1 [35]–[37] as well as hHO-1 in complex with imidazole-based inhibitors [26], [27], [30], [31]. The hHO1-t233/pBAce expression plasmid was a generous gift from Dr. Ortiz de Montellano (University of San Francisco). Bacterial expression and purification of hHO-1 from DH5α cells, and subsequent heme conjugation, were performed as described previously [30], based on published protocols.

A truncated, soluble version of hHO-2 containing 264 amino acids was utilized for comparison studies with hHO1-t233. The hHO-2(1-264)/pET28a expression plasmid was a generous gift from Dr. Stephen Ragsdale (University of Michigan). The expressed protein contained an N-terminal histidine tag to allow purification by metal chelation. Briefly, BL21 (DE3) cells, transformed with the hHO-2(1–264)/pET28a plasmid, were grown in 1 L of LB supplemented with 30 µg/mL of kanamycin at 37°C following inoculation from a miniculture. After reaching an OD_600_ of ∼0.8, protein expression was induced with 1 mM IPTG in the presence of 50 mg 5-aminolevulinic acid hydrochloride (Sigma) (the latter was later deemed unnecessary). Cells were allowed to grow for a further 3–4 hours at 37°C before harvesting by centrifugation. Cell pellets were stored at −80°C until ready for use. Pellets were subsequently thawed on ice, resuspended in lysis buffer [50 mM NaH_2_PO_4_ (pH 8.0), 300 mM NaCl, 3 mM imidazole, lysozyme (0.5 mg/mL; BioShop), DNAseI (5 units/mL; Fermentas) EDTA-free protease inhibitor cocktail (1 tablet/50 mL; Roche)] and incubated on ice for 30 min with rocking, followed by sonication. Lysates were cleared by centrifugation and the supernatant incubated with Ni-NTA agarose resin, which had been equilibrated with 50 mM NaH_2_PO_4_ containing 300 mM KCl, for 1 hour at 4°C. Subsequently, the resin was poured into a column for purification. Unbound protein was washed with buffer containing 15 mM imidazole, followed by a gradient from 20–200 mM imidazole; the protein eluted with 500 mM imidazole. Protein-containing fractions were visualized by 12% SDS-PAGE, pooled and dialyzed against 50 mM Tris pH 7.5, 100 mM KCl. The apo protein concentration was determined using the Bradford method (BioRad). A stock hemin (Fluka) solution (8 mg/mL) was prepared by dissolving 12 mg into 170 µL of 10% ethanolamine, followed by dilution into 50 mM Tris (pH 7.5), 100 mM KCl. The hemin solution was slowly added to the apo hHO-2 protein preparation to a final molar ratio of 2∶1, rocked at 4°C for 20–30 min, and stored at −20°C. Subsequently, excess heme was removed by passage over a PD-10 size-exclusion column (Amersham Biosciences) that had been equilibrated with 50 mM Tris (pH 7.5), 100 mM KCl. Protein concentration was determined by absorbance (ε405) 171.4±1.2 mM^−1^ cm^−1^) [46]. Purity was assessed by measurement of the Rz ratio (A_405_/A_280_>2.1) and by SDS-PAGE analysis.

#### Spectral Analysis

Inhibitor binding and heme degradation assays were performed on recombinant hHO proteins by measuring absorbance using a microplate spectrophotometer (Bio-Tek Instruments) as described previously [30], [33]. Briefly, inhibitor binding was determined by incubation of 10 µM heme-conjugated hHO-1 with increasing concentrations of **QC-308** (0–25 µM) in 20 mM potassium phosphate buffer. Spectra were recorded between 300 and 700 nm at 1 nm intervals. After the determination of the Soret peak position at each inhibitor concentration, heme degradation was initiated by the addition of l-ascorbic acid to a final concentration of 1 mM and followed by measuring absorbance at the wavelength corresponding to the respective Soret peaks at 1 min intervals for a period of 90 min. Initial rates of decrease of the Soret band were calculated at the wavelengths of the respective Soret peaks. Spectra were measured from 300 to 700 nm following the reaction period.

#### Crystallization

Crystallization was performed using sitting-drop vapour diffusion as described previously [30], based on published protocols [35]–[37]. The crystallization conditions consisted 100 mM HEPES (pH 7.5), 2.2 M ammonium sulfate 0.95% 1,6-hexanediol. The heme–hHO-1 complex (412 µM in 20 mM potassium phosphate) was mixed with **QC-308** at a molar ratio of 1∶3 and binding was confirmed using spectral analysis to confirm a shift in the native Soret peak (from 404 nm to 410 nm) prior to setting up crystallization plates, as described previously [30]. Crystallization drops consisted of 2 µL of the protein-inhibitor solution mixed with 2 µL of the reservoir solution.

#### Data Collection and Structure Determination

X-ray diffraction measurements were performed at the Advanced Photon Source (APS) at Argonne National Laboratory (Chicago, Illinois) at the 23-ID-D beamline. For data collection, a cryoprotectant comprising 100 mM HEPES pH 7.5, 2.32 M ammonium sulfate, 1% 1,6-hexanediol, 20% glycerol was used. Crystals were subsequently flash-cooled in a stream of N_2_ at 100 K. Data were collected for 360° with an oscillation of 0.5° and 240 frames processed using XDS for structure determination [52]. The structure of the protein-inhibitor complex was solved by molecular replacement (MR) using Phaser [53] in the CCP4 suite [54]. The heme-hHO-1 complex (PDB code 1N3U, Chain A) was the initial probe. An initial template of **QC-308** was generated using the Dundee PRODRG2 server [55], and the library files created using Monomer Sketcher in the CCP4 suite. Following initial refinement with Refmac in the CCP4 suite, the structure of the inhibitor was inserted and subsequently refined using iterative cycles of Coot [56] and Refmac5 [57] in the CCP4 suite. Standard parameters for heme were used as described in its library entry in the program during refinement, with no additional restraints on planarity, bond lengths, and bond angles. Structural alignments were performed using Superpose [58] in CCP4. Contacts were calculated using Contacts in CCP4. Ramachandran plots were determined using PROCHECK in CCP4 [59]. Electrostatic surface potentials were calculated and all images were prepared using PyMOL [60].

## Supporting Information

Figure S1 **Structure of heme-conjugated hHO-1 in complex with 1-(1** ***H*** **-imidazol-1-yl)-4,4-diphenyl-2-butanone (QC-308) at 2.85 Å resolution.** Ribbon diagram of the two molecules in the asymmetric unit. Heme (orange) and **QC-308** (yellow) are depicted as stick models, as is the molecule of 1,6-hexanediol (magenta). Image was created in PyMol [60].
(TIF)

Figure S2 **Structural alignment.** Top: Plot of the RMS deviation of main chain backbone atoms of the A and B molecules of hHO-1 in complex with **QC-308**. Bottom: Alignment of the B molecule with the native holoenzyme (PDB code 1N45, Chain-A), hHO-1 in complex with **QC-82** (PDB code 3CZY, Chain-A), and hHO-1 in complex with **QC-80** (PDB code 3HOK, Chain B). Structural alignments were performed using “Superpose” in CCP4 [54], [58].
(DOC)

Table S1 **Contacts between heme-conjugated hHO-1 and 1-(1** ***H*** **-imidazol-1-yl)-4,4-diphenyl-2-butanone.** Residues ≤4.0 Å apart are listed. Atom names of inhibitor refer to nomenclature in the PDB. Distances were calculated using “Contacts” in CCP4 [54].
(DOC)
