# Supplementary figures and images for: A Novel, “Double-Clamp” Binding Mode for Human Heme Oxygenase-1 Inhibition
Source: PLoS One. 2012 Jan 19;7(1):e29514. doi: 10.1371/journal.pone.0029514 (PMC3261875; doi:10.1371/journal.pone.0029514)

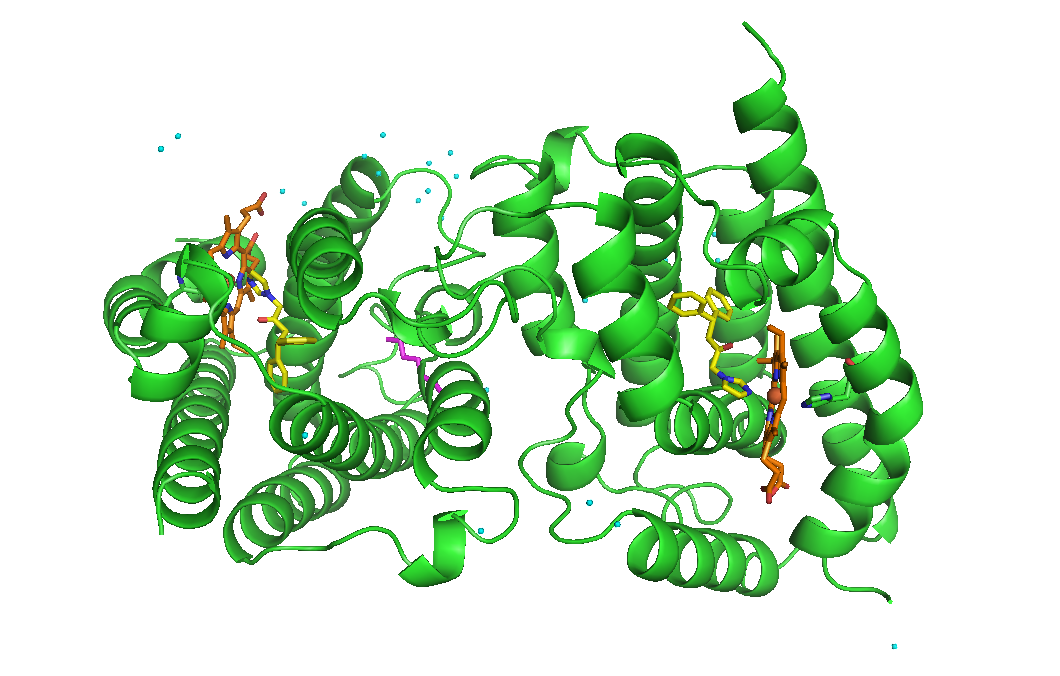

Supplement: Figure S1 — Structure of heme-conjugated hHO-1 in complex with 1-(1 H -imidazol-1-yl)-4,4-diphenyl-2-butanone (QC-308) at 2.85 Å resolution. Ribbon diagram of the two molecules in the asymmetric unit. Heme (orange) and QC-308 (yellow) are depicted as stick models, as is the molecule of 1,6-hexanediol (magenta). Image was created in PyMol [60]. (TIF) [file pone.0029514.s001.tif]

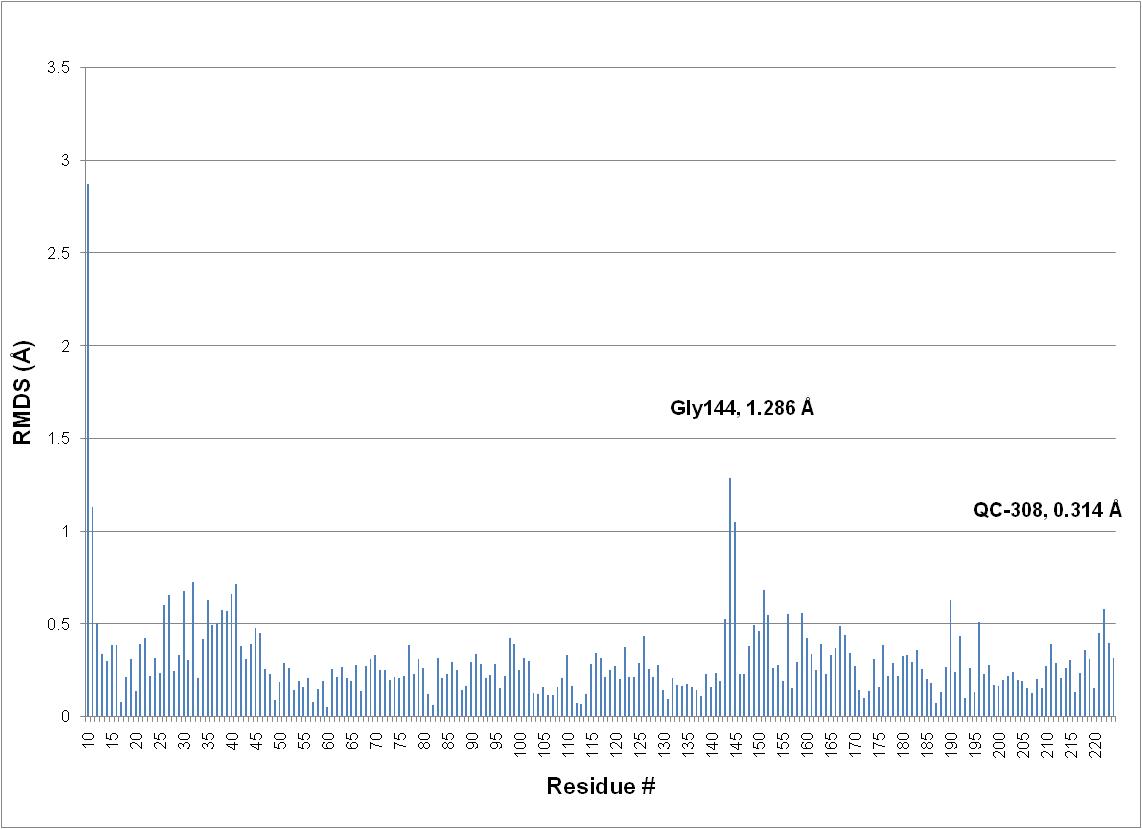


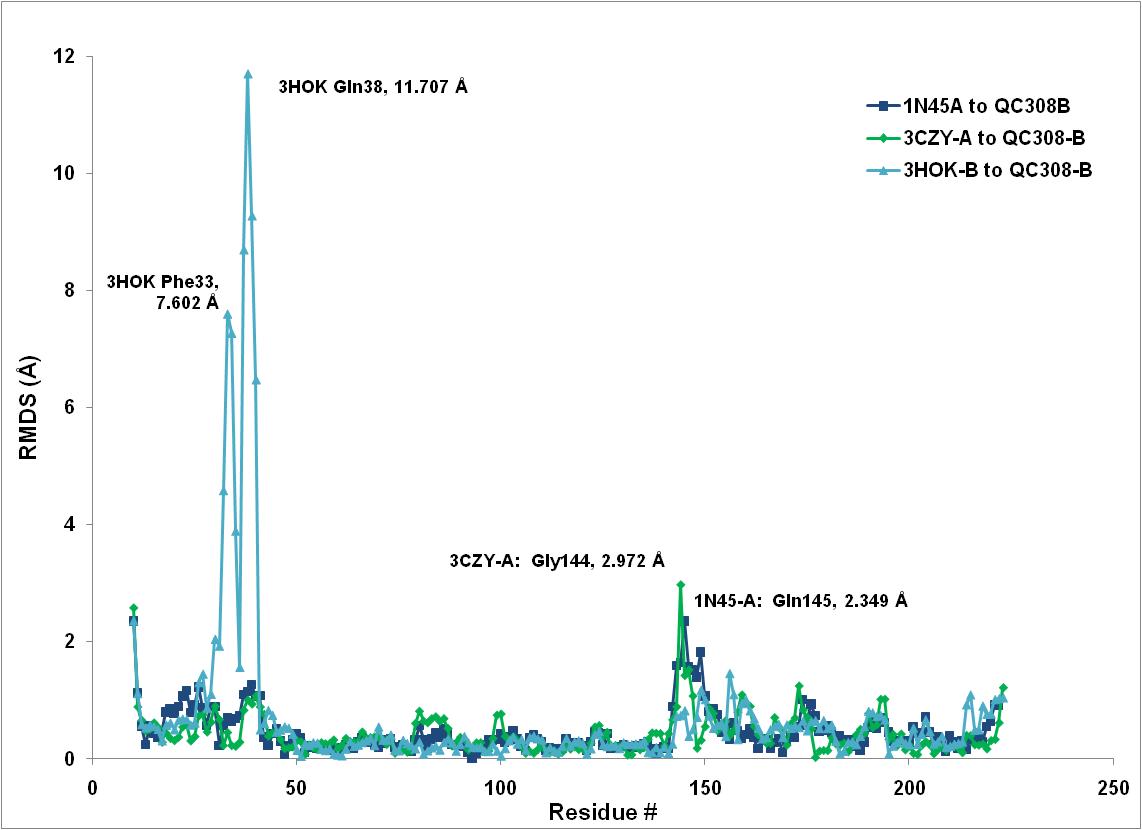

Supplement: Figure S2 — Structural alignment. Top: Plot of the RMS deviation of main chain backbone atoms of the A and B molecules of hHO-1 in complex with QC-308. Bottom: Alignment of the B molecule with the native holoenzyme (PDB code 1N45, Chain-A), hHO-1 in complex with QC-82 (PDB code 3CZY, Chain-A), and hHO-1 in complex with QC-80 (PDB code 3HOK, Chain B). Structural alignments were performed using “Superpose” in CCP4 [54], [58]. (DOC) [file pone.0029514.s002.doc]
