# Supplementary material for: A Novel, “Double-Clamp” Binding Mode for Human Heme Oxygenase-1 Inhibition
Source: PLoS One. 2012 Jan 19;7(1):e29514. doi: 10.1371/journal.pone.0029514 (PMC3261875; doi:10.1371/journal.pone.0029514)
Supplement: Table S1 — Contacts between heme-conjugated hHO-1 and 1-(1 H -imidazol-1-yl)-4,4-diphenyl-2-butanone. Residues ≤4.0 Å apart are listed. Atom names of inhibitor refer to nomenclature in the PDB. Distances were calculated using “Contacts” in CCP4 [54]. (DOC) [file pone.0029514.s003.doc]

**Table S1.** Contacts between heme-conjugated hHO-1 and 1-(1*H*-imidazol-1-yl)-4,4-diphenyl-2-butanone.

| **Inhibitor** | **Heme–hHO-1** | | **Distance (Å)** |
| --- | --- | --- | --- |
| O | Heme | CHC | 3.9 |
|  |  | C1C | 4.0 |
| C | Asp140 | OD1 | 3.8 |
| CA | Gly139 | C | 3.9 |
|  |  | O | 3.4 |
|  | Asp140 | CA | 4.0 |
|  |  | OD1 | 3.6 |
|  | Leu147 | CD1 | 3.6 |
| N | Gly139 | C | 3.8 |
|  |  | O | 3.1 |
| CAN | Gly139 | C | 3.9 |
|  |  | O | 3.4 |
|  | Heme | N­_B | 3.8 |
|  |  | CHC | 3.9 |
|  |  | CHD | 4.0 |
|  |  | C1C | 3.5 |
|  |  | C4C | 3.5 |
|  |  | N_C | 3.0 |
|  |  | N_D | 3.6 |
|  |  | Fe | 3.0 |
| NAQ | Gly139 | O | 3.8 |
|  | Heme | CHA | 3.9 |
|  |  | C1A | 3.6 |
|  |  | C4A | 3.8 |
|  |  | N_A | 3.0 |
|  |  | C1B | 3.8 |
|  |  | C4B | 3.7 |
|  |  | N_B | 3.0 |
|  |  | CHC | 3.9 |
|  |  | C1C | 3.7 |
|  |  | C4C | 3.7 |
|  |  | N_C | 2.9 |
|  |  | C4D | 3.7 |
|  |  | N_D | 2.9 |
|  |  | Fe | 2.1 |
|  |  | C1D | 3.8 |
| CAH | Gly139 | O | 3.8 |
|  | Gly143 | N | 4.0 |
|  | Heme | C1A | 3.7 |
|  |  | C4A | 3.6 |
|  |  | N_A | 3.2 |
|  |  | CHB | 4.0 |
|  |  | C1B | 3.8 |
|  |  | N_B | 3.5 |
|  |  | N_D | 4.0 |
|  |  | Fe | 3.1 |
| CAM | Gly139 | O | 3.3 |
|  | Gly143 | N | 3.4 |
|  |  | CA | 3.4 |
|  | Leu147 | CD1 | 4.0 |
| CAO | Asp140 | OD1 | 3.2 |
| CAJ | Val50 | CG1 | 4.0 |
| CAE | Phe167 | CE1 | 3.5 |
|  |  | CZ | 3.5 |
|  | Val50 | CG1 | 3.7 |
| CAB | Phe167 | CE1 | 4.0 |
|  |  | CZ | 3.7 |
|  | Phe47 | CE1 | 4.0 |
|  | Val50 | CG1 | 3.8 |
| CAD | Phe37 | CE2 | 3.9 |
|  |  | CZ | 3.8 |
| CAI | Met34 | CE | 3.4 |
| CAT | Phe214 | CZ | 3.8 |
| CAL | Leu54 | CD2 | 3.9 |
|  | Val50 | CG1 | 3.7 |
|  | Phe214 | CE1 | 3.9 |
| CAG | Leu54 | CG | 4.0 |
|  |  | CD2 | 3.7 |
|  | Phe214 | CE1 | 3.8 |
| CAC | Arg136 | NH2 | 4.0 |
|  | Leu213 | CD2 | 3.8 |
|  | Phe214 | CE1 | 3.8 |
|  |  | CZ | 3.8 |
|  | Asn210 | OD1 | 3.6 |
| CAF | Arg136 | NH1 | 3.7 |
|  |  | NH2 | 3.2 |
|  |  | CZ | 3.8 |
|  | Phe214 | CZ | 3.5 |
|  |  | CE1 | 4.0 |
|  | Asn210 | CG | 3.9 |
|  |  | OD1 | 3.0 |
| CAK | Arg136 | NH2 | 3.3 |
|  | Phe214 | CZ | 3.5 |
